# Utilization and cost of electronic resources in adult cancer center

**DOI:** 10.1186/s13104-018-3637-6

**Published:** 2018-07-28

**Authors:** Fatma Maraiki, Siobhan Kelly, Mohamed Ahmed, Sakra Balhareth, Tusneem Elhasan, Mahmoud Aljurf, Shouki Bazarbashi

**Affiliations:** 1 King Faisal Specialist Hospital and Research Center, P.O.BOX 3354, Riyadh, 11211 Saudi Arabia; 2 Mater Private Hospital, Eccles Street, Dublin 7, Ireland

**Keywords:** Electronic, Institution, Resources, Utilization, Awareness, Cost, Cancer Center

## Abstract

**Objectives:**

This study aims to evaluate the knowledge of healthcare providers and the cost of the current institutional e-resources in an adult oncology setting. To assess the awareness, accessibility, and utilization of the available intranet e-resources, a survey questionnaire was distributed to all oncology healthcare practitioners (physicians, nurses, and pharmacists) in an adult oncology center. The e-resources were divided into two main categories: pre-paid and institution-specific. The cost of the pre-paid e-resources was obtained from the relevant department. The cost of the institution-specific e-resources was calculated based on the human cost spent developing these e-resources; the cost of the information technology (IT) and the organizational overhead were also taken into consideration.

**Results:**

Institution-specific e-resources constituted the majority (62%) versus (38%) for pre-paid. The overall awareness, access, and frequent utilization of institution-specific e-resources, as compared to pre-paid e-resources, were low (< 50%). The cost of the institution-specific e-resources was $1,137,196, which was more than ten times higher than the pre-paid e-resources. This study identifies the general lack of awareness and utilization of institutional e-resources. The low utilization coupled with the high cost of the institution-specific e-resources makes pre-paid e-resources an attractive alternative for any institution.

## Introduction

Electronic resources (e-resources) broadly include all systems used to aid learning, administration, or management in a given institution. Such e-resources can be accessed through a hospital intranet. The strategic information management of e-resources is vital in any healthcare institution to promote awareness, allow the exchange of information, and provide cost-effective information technology [1, 2]. The utilization of such e-resources ensures that practice is unified and it should ultimately enhance patient care and safety as well. Institution-specific e-resources are typically exclusive to a given institution and access is frequently restricted to authorized clients only. These e-resources include either purchased commercial resources (pre-paid) or institution-specific resources that have been formulated by employees within the institution—through collaborative initiatives, committees, and/or taskforce meetings. These e-resources may include (but are not limited to) policies and procedures, guidelines, quick reference tables, and charts. Institutions have a duty, through a dedicated informatics team, to continuously promote the awareness, ensure easy access, and avoid fragmentation and duplication of information [3, 4]. Numerous resources are typically available to healthcare institutions to allow the sharing of information with employees. However, little information is available to inform as to which of these e-resources are the most widely utilized or provide the best value for money.

This study aims to evaluate the healthcare providers’ knowledge of the current institutional e-resources in an adult oncology setting. The cost of managing and providing the e-resources will also be calculated. The purpose of the cost calculations is not to determine the cost-effectiveness, but rather to illuminate the relative cost and time incurred in creating healthcare institution-specific e-resources.

## Main text

### Methods

A survey questionnaire was developed, validated, and distributed to all of the oncology healthcare practitioners (physicians, nurses, and pharmacists) in an adult oncology center. The questionnaire consisted of three sections, which assessed the awareness, accessibility, and utilization of the available intranet e-resources. The questionnaire was distributed to 293 practitioners, which consisted of 66 physicians, 200 nurses, and 27 pharmacists. Following the data collection, the investigators undertook a proactive approach in educating the practitioners who were either unaware of the availability or who had difficulty in accessing any of the e-resources. The team calculated the proportion of the healthcare professionals in the oncology center who were aware/accessed/utilized the available e-resources. Data were collected as a total percentage of the whole groups, without stratification of the different professions.

The investigators evaluated the intranet content to include all of the e-resources relevant to oncology practice. It must be noted that some of the evaluated e-resources were not purely oncology specific, but were still included in the data since they contained some oncology related content. All e-resources were sorted under two categories: medication and disease. These were further sub-classified into two categories: pre-paid and institution-specific. Institution-specific e-resources are those formulated by the institution through collaborative initiatives, committees, and/or taskforce meetings. The final draft of these e-resources are presented in a portable document format (PDF) documents, which are then uploaded onto the hospital intranet. The medication related category consisted of two major pre-paid e-resources: Lexicomp^®^ and Micromedex^®^, which are collations of clinical database and clinical decision support tools. The only pre-paid e-resources under disease category is UpToDate database, which is a clinical decision support resource that covers a wide range of disease management. While UpToDate is used extensively across the institution by other specialties, it is considered a vital e-resource for oncology practice.

The cost of the pre-paid e-resources (Lexicomp, Micromedex, UpToDate) were obtained from the relevant departments and were presented as a total cost. This was done in order to maintain the confidentiality of the purchasing cost of individual pre-paid e-resources to the institution.

The cost of the institution-specific e-resources was calculated by the Financial Affairs Department and was based on the following criteria:

1. Human cost: the time spent on these activities.
2. Information technology (IT) costs: the specific IT solutions/systems used.
3. Organizational overhead: the infrastructure costs incurred.

The majority of the institution-specific e-resources assessed in this study relate specifically to institutional medication guidelines. These guidelines are produced by an Oncology Subcommittee under the remit of the hospital Pharmacy & Therapeutics Committee (P&T). Consequently, the cost of the institution-specific e-resources is an indirect reflection of the cost of the time spent for establishing these e-resources by the oncology subcommittee of the P&T.

### Statistical considerations

This is a descriptive study that aims to evaluate the utilization of the available hospital e-resources. A questionnaire was used to measure the hospital’s e-resource utilization. The data were described in term of frequencies and percentages.

## Results

A total of 141 practitioners responded to the survey, a 59% response rate. The response rate among pharmacists, physicians, and nurses, was 92, 50, and 44%, respectively. Staff from in-patient, out-patient, and combined practice settings were evenly represented in the survey. The majority of the participants (50.3%) had less than 5 years of experience.

As shown in Table 1, the three main pre-paid e-resources were Micromedex, Lexicomp, and UpToDate. The awareness of the pre-paid e-resources was 93% for Lexicomp, 70% for UpToDate, and 57% for Micromedex. The access was almost similar to the awareness, with 90% of the participants able to access Lexicomp, 66% UpToDate, and 50% Micromedex. Overall, the utilization of the pre-paid e-resources was 60, 31, and 16% of the participants stating that they frequently utilized Lexicomp, UpToDate, and Micromedex, respectively. The overall awareness, access, and frequent utilization of institution-specific e-resources, as compared to pre-paid e-resources, were low. Less than 50% of the respondents were aware of, able to access, or frequently utilized institution specific e-resources.

**Table 1:** Survey

	Aware of (%)	Can access (%)	Utilization (%)		
			Never	Occasional	Frequent
Medication related e-resources					
Pre-paid e-resources					
Micromedex	57	50	51	33	16
Lexicomp with integrated institutional formulary	93	90	10	30	60
Institution-specific e-resources					
Chemotherapy preparation chart	40	34	69	17	14
List of available non-formulary and investigational drugs	49	44	61	31	8
Chemotherapy dose adjustment guidelines for renal and hepatic dysfunction	35	27	75	17	8
Institutional carboplatin dosing guidelines, other than formulary	37	31	71	20	9
Formulary oncology medication prescribing guidelines	64	60	42	33	25
Extravasations management reference table	53	46	60	32	8
Chemotherapy-induced nausea and vomiting guidelines	51	45	60	23	17
Disease related e-resources					
Pre-paid e-resources					
UpToDate	70	66	35	34	31
Institution-specific e-resources					
Institutional cancer site treatment guidelines	47	39	59	24	17

When asked about the ease of access and the utilization of available e-resources, only 56.4% of the respondents believed that the available intranet e-resources were clear, 42% that they were appropriately categorized, and 52% that they were user friendly, see Table 2. While only 35% of the respondents said that the available e-resources were introduced during training sessions, the majority of the respondents, 87.5%, recognized the importance of these e-resources for their daily professional practice.

**Table 2:** Awareness or access/utilization issues

Access/utilization issues	Percentage
Access to the intranet e-resources is clear	56.4
Access to the intranet e-resources is appropriately categorized	42
Access to the intranet e-resources is user-friendly	52
Access to the intranet e-resources was introduced during the hospital/departmental orientation	35
Sending alerts/emails for newly uploaded material would encourage to access and utilize the e-resources	77.8
The survey e-resources are required for your practice/professional activities	87.5

The cost of the institution-specific e-resources was $1,137,196, which was more than ten times higher than the pre-paid e-resources (Table 3).

**Table 3:** Cost analysis

Annual cost ($)	Pre-paid e-resources	Institution-specific e-resources
Human cost	Negligible	921,005
Information technology cost	Negligible	Negligible
Overhead cost	Not applicable	216,191
Purchasing cost	114,666	Not applicable
Total cost	114,666	1,137,196

## Discussion

Our survey demonstrated that the overall utilization of the hospital’s e-resources (institutional and pre-paid), was low. It was noteworthy that institution-specific e-resources represented the lowest figure of utilization, as compared to pre-paid e-resources.

The overall survey response rate was 59% of the total sample. The higher response rate from pharmacists, in comparison to nurses and physicians was expected, since pharmacists have a greater role in establishing, uploading, and training staff about the available e-resources. Nonetheless, pharmacists only constituted 11% of the respondents and this may have resulted in lower figures regarding the awareness, access, and utilization of e-resources in the overall study. Half of the participants had less than 5 years of experience in the institution, which reflects the high turnover of staff within the hospital. This may also have contributed to the low overall awareness for the institutional e-resources and it highlights the need for continuous training as well.

Lexicomp was the most widely accessed and the most frequently utilized e-resource. Since the institutional formulary is integrated within Lexicomp, the widespread use of Lexicomp was an expected outcome. Furthermore, Lexicomp is located in a prominent position on the homepage of the hospital intranet. In comparison, only half of the respondents were aware of or accessed/utilized Micromedex. This may be related to the fact that the location of Micromedex on the hospital intranet is somewhat obscure. UpToDate was accessed frequently and it was utilized by the respondents; over two-thirds had either frequent or occasional utilization.

In general, the awareness and access of the institution-specific e-resources by the respondents was poor. The massive underutilization of institution-specific e-resources was the most striking finding; up to 75% of the respondents had never utilized these e-resources. The most troublesome aspect of this finding was that most of these institution-specific e-resources are essential for day-to-day practice within the oncology center. A possible explanation for the underutilization of the institution-specific e-resources is the fact that the link to the oncology center through the intranet homepage is not easily identifiable.

The findings of our study are in line with those of a similar study that evaluated the knowledge and use of electronic information e-resources by a medical faculty, which had a high overall awareness but low utilization [5]. One possible solution to increase the utilization of the institution-specific e-resources would be integration with the hospital computer provider order entry (CPOE) system, as a medication-related clinical decision support tool [6–8].

Only half of the respondents stated that the intranet e-resources were clear, appropriately categorized, and user-friendly. This suggests that the available e-resources were inaccessible, fragmented, and not user friendly. Studies have shown that simplified e-resources can improve the performance and experience of end users [9, 10]. Nearly two-thirds of the respondents stated that e-resources were not addressed during general hospital training sessions. This highlights the need for continuous and structured training to allow practitioners to utilize e-resources effectively and efficiently, particularly in a specialized setting such as oncology. The lack of formal training on the use of e-resources is a well-recognized problem and can lead to underutilization of e-resources.

The cost of the institution-specific e-resources was more than ten times higher than that of the pre-paid e-resources. The actual cost of the institution-specific e-resources indirectly reflects part of the costs of the committees responsible for producing them. This illuminates the cost of the major committees in any hospital.

## Conclusion

This study identifies the general lack of awareness and utilization of institutional e-resources in an adult oncology center. The major barrier was the accessibility of these e-resources, which highlights the need for institutions to have an easily accessible, concise, and user-friendly intranet. The low utilization coupled with the high cost of the institution-specific e-resources makes pre-paid e-resources an attractive alternative for any institution.

## Limitation

One of the main limitations of our study is that it is a single center study and its general findings may not be germane to other institutions.

## Abbreviations

E-resources: electronic resources
IT: information technology
P&T: Pharmacy and Therapeutic Committee
CPOE: computer provider order entry
